# Video-Based Behaviorally Coded Movement Assessment for Adolescents with Intellectual Disabilities: Application in Leg Dribbling Performance

**DOI:** 10.3390/s23010179

**Published:** 2022-12-24

**Authors:** Hsin-Yi Kathy Cheng, Wann-Yun Shieh, Yu-Chun Yu, Pao-Wen Li, Yan-Ying Ju

**Affiliations:** 1 Graduate Institute of Early Intervention, College of Medicine, Chang Gung University, No. 259, Wen-Hwa 1st Road, Kwei-Shan, Tao-Yuan 333, Taiwan; 2 Department of Physical Medicine and Rehabilitation, Chang Gung Memorial Hospital, 5 Fu-Hsing Street, Kwei-Shan, Tao-Yuan 333, Taiwan; 3 Department of Computer Science and Information Engineering, College of Engineering, Chang Gung University, No. 259, Wen-Hwa 1st Road, Kwei-Shan, Tao-Yuan 333, Taiwan; 4 Taoyuan Municipal Taoyuan Special School, No. 10, Deshou Street, Taoyuan District, Tao-Yuan 330, Taiwan; 5 Department of Adapted Physical Education, National Taiwan Sport University, No. 250, Wen-Hwa 1st Road, Kwei-Shan, Tao-Yuan 333, Taiwan

**Keywords:** intellectual disability, motor performance, lower extremity, video-based, wearable

## Abstract

Measuring motor performance in individuals with intellectual disabilities (ID) is quite challenging. The objective of this study was to compare the motor performances of individuals with ID and those with typical development (TD) during soccer dribbling through video-based behavior-coded movement assessment along with a wearable sensor. A cross-sectional research design was adopted. Adolescents with TD (N = 25) and ID (N = 29) participated in the straight-line and zigzag soccer dribbling tests. The dribbling performance was videotaped, and the footage was then analyzed with customized behavior-coding software. The coded parameters were the time for movement completion, the number of kicks, blocks, steps, the number of times the ball went out of bounds, the number of missed cones, and the trunk tilt angle. Participants with ID exhibited significantly poorer performance and demonstrated greater variances in many time and frequency domain parameters. It also revealed that participants with ID kicked with both feet while dribbling, whereas those with TD mainly used the dominant foot. The present findings demonstrated how the ID population differed from their peers in lower-extremity strategic control. The customized video-based behavior-coded approach provides an efficient and effective way to gather behavioral data and calculate performance parameter statistics in populations with intellectual disabilities.

## 1. Introduction

Adolescents with intellectual disabilities (ID) exhibit relatively unstable and variable movement characteristics. Their motor impairments include deficiencies in the aspects of motor control, such as coordination, reaction time, agility, balance control, muscle strength, and endurance, and they may exhibit comorbidities such as attention deficit hyperactivity disorders or developmental coordination disorders [1,2,3,4,5,6,7,8,9,10]. Consequently, they are less fit than the typically developing (TD) peers and often have difficulties engaging in movements such as walking and running [1,2,11,12].

The movement of adolescents with ID can be evaluated using regular gross motor tests or sensors [3,13,14,15]. However, some tests are not sensitive enough to differentiate TD adolescents from those with ID or are hard to set up with non-collaborative users. Shieh et al. (2016) found that adolescents with ID present significant differences in locomotor performance compared with adolescents with TD only when the test is more challenging. Iosa et al. (2014) reported no significant difference between these two groups in straight-line walking unless the participants have multiple disabilities. Furthermore, most participants with ID fail to follow the instructions or stay focused unless the task is familiar or fun. Therefore, tracking the movement performance in individuals with ID is relatively challenging.

Soccer games are often adopted by schools and institutions for group play and social exchanges because they are entertaining, easy to learn, and promote interaction and physical activity [16]. It is also one of the sports offered by Special Olympics designed for athletes with conditions such as Down Syndrome whose ability levels have not yet prepared them for team play [17]. Effective dribbling requires players to have basic motor control, including dynamic balance, agility, eye-foot coordination, and muscular endurance [17,18,19,20]. By observing the time and frequency domain parameters during soccer dribbling, the movement limitations within the individuals with ID can be identified.

Recent technologies for movement analysis that leverage contact sensors have been developed [15,21]. However, adolescents with ID are sometimes non-collaborative; hence the application of contact sensors can be challenging. Markerless approaches are easier to set up, making them suitable technologies for individuals with ID. Three-dimensional motion capture is considered the gold standard for human movement analysis. However, it is expensive and requires a significant amount of time [22,23]. Rather, the video-based observation method, hereby meaning observation recorded via cameras with further image-registered behavior coding, requires little cooperation from the target individuals and is easier to set up. It can be used to record, register, and output pre-defined observed data for statistical analysis [24,25,26,27]. In this framework, the video-based behavior coding system can be combined with any wearable device to provide a more complete measurement of soccer performance in individuals with ID. Their dribbling performance can be assessed by analyzing the coded parameters from the video clips, along with the sensor patterns on their body parts.

Therefore, the purpose of this study was to explore the differences in the lower extremity strategic control during dribbling movements between adolescents with ID and their TD peers via video-based observation with customized behavior coding along with a wearable sensor to monitor trunk behavior. We hypothesized that adolescents with ID would perform differently in the time and frequency domain and make more mistakes than their TD peers.

## 2. Materials and Methods

In this study, we used 2D video cameras (Sony Handycam CX-240, Sony Taiwan, Taipei, Taiwan) to record the dribbling performance of the participants. The video clips were then analyzed using the behavior coding and analysis software (Observer XT 10, Noldus Information Technology, Leesburg, VA, USA). The dribbling behaviors were coded in a quantitative way and can be visualized on a timeline. Descriptive statistics were reported to describe the basic features of the data, and inferential statistics were used to identify the differences between the two groups. Details are shown below.

### 2.1. Participants

Twenty-Nine adolescents between the age of 16 to 19 years with moderate to severe ID and 25 of their TD peers were recruited from a special education school and a local senior high school. Those who were unable to walk, follow simple instructions, had a diagnosis of apparent orthopedic diseases or bone fractures within six months prior to the study, and currently received regular soccer training or enrollment in a soccer program were excluded. All the participants and their guardians provided their consent before participating in this study and were fully informed of the experiment prior to commencement. The research design received approval from the institutional review board in Chang Gung Memorial Hospital (IRB No. 103-7262B). All participants were right-leg dominant.

### 2.2. Instruments

**Figure 1:**
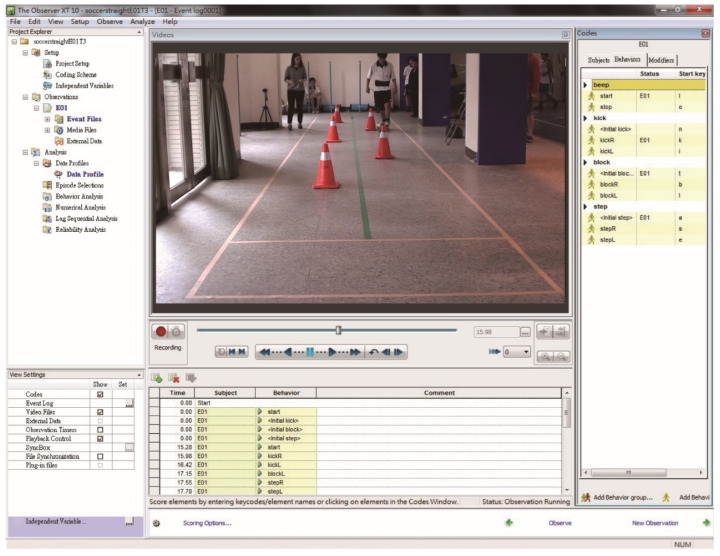
The software interface and the flow grid of the testing instrument.

The frame rate of the camera used in the experiment was 25 Hz [28,29,30]. The Observer XT was used for analyzing, managing, and presenting observational data. After the formulation of the coding scheme, real-time behavior coding was conducted along with the events that occurred in the video, and the software output designated data for statistical analysis. The software interface is shown in Figure 1. It can also provide the time at which a motor behavior occurs, the duration of the motor event, and quantitative data regarding the motor events.

**Figure 2:**
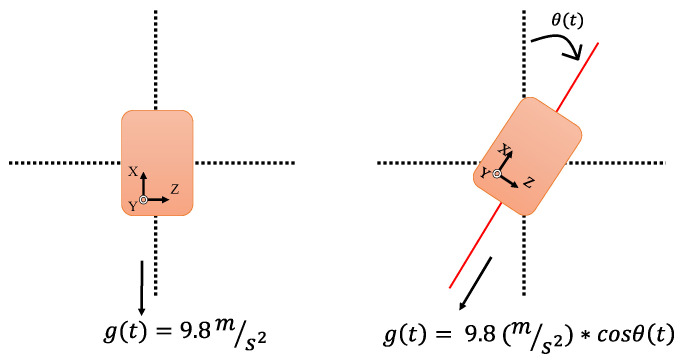
Adjusting gravity influence via angle θ(t) correction.

In addition, an Xsens MTw motion tracker (Xsens Technologies, Enschede, The Netherlands) was used to measure the trunk motion in the sagittal and frontal planes. The sampling rate was 1 kHz. Since the output of the accelerometer would be influenced by gravity, especially during dribbling, the authors managed to eliminate the influences of gravity and drift:

(1) $$a_{x}\left(t\right)=a_{x}’\left(t\right)-\left[9.8*cos\theta \left(t\right)\right].$$

where $a_{x}\left(t\right)$ is the free acceleration of the sensor along the *X*-axis, $a_{x}{}^{’}\left(t\right)\;$is the output of the accelerometer along the *X*-axis, and *g(t)* is the gravitational acceleration. The function of *g(t)* will have a component on the *X*-axis, depending on the angle of the tracker rotated. If the sensor rotates clockwise around the *Y*-axis by angle *θ(t)*, as shown in Figure 2, then we have $g\left(t\right)=9.8\left(m/s^{2}\right)*cos\theta \left(t\right)$. θ(t) in Equation (1) can be obtained by integrating the angular velocity (rad/s) from the gyroscope. The drift of the gyroscope signals should be corrected before data integration. The maximum excursion was calculated. Here we use the complementary filter to remove the drift from the gyroscope, and the details of signal processing can be found in our previous work [15,31].

### 2.3. Coded Parameters

The video clips were used for analysis. The flow grids of the system allowed the registrations to be made over time: the presence, frequency, duration, and value of each designated behavior. The coded behavior variables are defined in Table 1. The authors needed to specify the keycodes, including the “start” and the “stop”, and the key features in dribbling events such as “kickL”, “kickR”, “blockL”, “blockR” and so on. L and R indicate left or right leg. These key features can be defined as independent variables with desired value ranges. To start scoring, the raters should first align the video to the particular time frame and register a particular event by typing the predefined key code. With multiple trials to go through, an auto record can be selected to score the same subject for consecutive events. One can go through the operation manual for specific coding details.

### 2.4. Procedure

**Figure 4:**
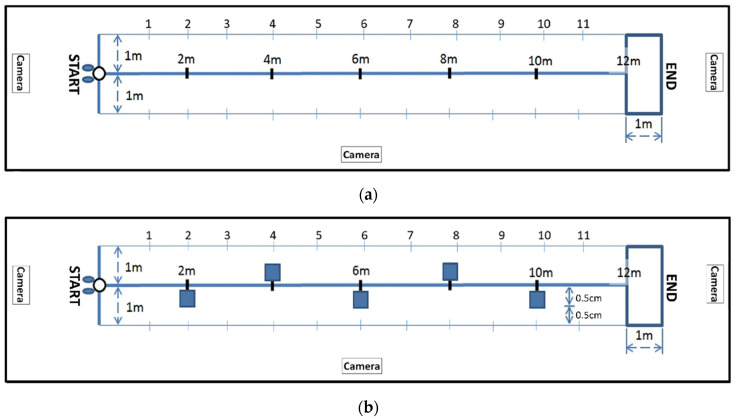
(**a**)The test site for straight-line dribbling and the location of video cameras; (**b**)The test site for zigzag dribbling and the location of video cameras.

The recording site was set up according to the Special Olympics individual soccer skills competition setting. The site used for straight-line dribbling measured 2 m × 12 m. A marking point was established every 2 m, and the final 1 m was designated as the finishing line where players could stop dribbling. Three cameras were installed surrounding the site to record participants’ dribbling (Figure 4a). The site used for zigzag dribbling also measured 2 m × 12 m, with two cones placed side by side every 2 m. Similarly, the final 1 m was designated as the finishing line where players could stop dribbling, and three cameras were installed surrounding the site to record participants’ dribbling (Figure 4b). The videos from three cameras were uploaded to the behavior-coding software and aligned along the same timeline. The first coded event of each video was taken as the synchronization point. All data files can be played synchronously. When a particular event is registered in the recording from the front camera, the rater can check the event occurrence from the other cameras simultaneously. If there was a blockage from the ball, extremity, or cones of the recording from the front camera, the rater could check the recording from the side or the back camera to register the movement event. To ensure the familiarity and convenience of the site, we conducted the study at the participants’ school. In addition, to ensure participant safety and minimize site changes, we conducted the test in an indoor setting with considerable room for movement and cleared all objects surrounding the site.

**Figure 5:**
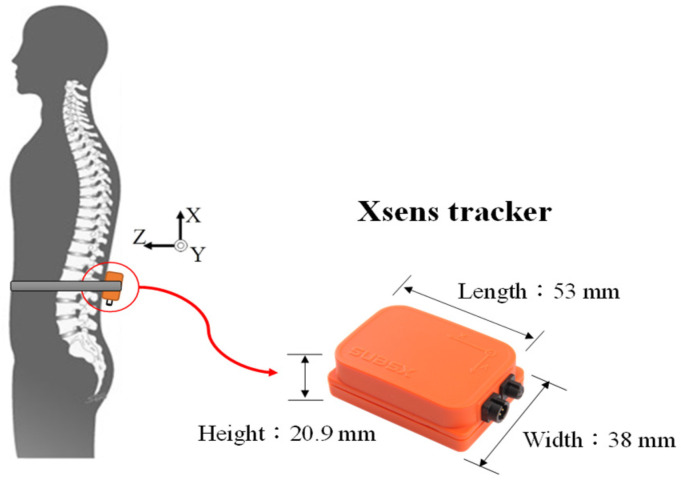
The tracker position of the participant (from Shieh et al. (2016)).

An Xsens MTw tracker was secured to the lower back for motion detection. The tracker was 47 × 30 × 13 mm^3^ in size and weighed 16 g. The signals were collected and stored by a laptop for further analysis. Figure 5 illustrates the tracker position on the lower back of the participant.

Following a warm-up and a demonstration, the participants practiced straight-line dribbling one time. A straight-line dribbling test was conducted first. The test began as the whistle blew and ended as the participant dribbled the ball into the finishing area. The participant then rested for one minute then engaged in the second and then the third straight-line dribbling test. Subsequently, the participants performed zigzag dribbling tests by following the same procedure. After completing all tests, the participants engaged in a 3-min cool-down. TD participants performed the same trials under the same conditions as IDs. A total of three straight-line and three zigzag dribbling tests were completed and analyzed. All the straight-line and zigzag dribbling tests were videotaped. Two raters examined the video clips and registered the events with the Observer XT 10 software. Since this was a semi-automatic procedure, the two-rater coding was to ensure the extent to which raters code the same units of data in the same way. When a statistical analysis was performed using SPSS22 descriptive statistics were sued to analyze participants’ demographic data. An independent samples t-test was conducted to compare the dribbling parameters of participants with ID and their typically developing peers. The significance level was set at *p* < 0.05.

## 3. Results

A total of 54 adolescents (29 ID and 25 TD) participated (Table 2). No significant differences were found in their age (*p* = 0.561), gender (*p* = 0.685), and height (*p* = 0.768). This study explored how the participants dribbled a soccer ball in the straight-line and zigzag dribbling sessions. Table 3 is a coding summary of a participant with ID performing zigzag dribbling. Results for the various parameters are summarized in Table 4. The inter-rater reliability was assessed based on the individual parameters without separating for each dribbling task. The inter-rater reliabilities for the individual dribbling parameters ranged from 0.87–1.

### 3.1. Total Time (in Seconds)

The ID and TD groups exhibited statistically significant differences in the total time spent in straight-line dribbling (*p* < 0.001) and zigzag dribbling (*p* < 0.001). Participants with ID spent more time dribbling than their typically developing peers.

### 3.2. Number of Out of Bounds

The ID and TD groups exhibited statistically significant differences in the number of out-of-bounds occurrences in straight-line dribbling (*p* = 0.001) and zigzag dribbling (*p* < 0.001). Specifically, participants with ID exhibited more out-of-bounds occurrences than their typically developing peers.

### 3.3. Number of Missed Cones

The ID and TD groups exhibited no significant differences in the number of missed cones in zigzag dribbling (*p* = 0.103); however, participants with ID exhibited more missed cones than their typically developing peers.

### 3.4. Total Number of Kicks

The parameters used to measure the total number of kicks were the total number of right-foot kicks, the total number of left-foot kicks, and the total number of kicks. During straight-line dribbling, the ID and TD groups exhibited statistically significant differences in the total number of right-foot kicks (*p* = 0.037), the total number of left-foot kicks (*p* = 0.009), and the total number of kicks (*p* = 0.002).

During zigzag dribbling, the ID and TD groups exhibited no significant difference in the total number of right-foot kicks (*p* = 0.110) but statistically significant differences in the total number of left-foot kicks (*p* = 0.036) and the total number of kicks (*p* = 0.049).

### 3.5. Total Number of Blocks

The total number of blocks were measured using the following parameters: the total number of right-foot blocks, the total number of left-foot blocks, and the total number of blocks. During straight-line dribbling, the ID and TD groups exhibited no significant difference in the number of right-foot blocks (*p* = 0.121) but statistically significant differences in the number of left-foot blocks (*p* = 0.004) and the total number of blocks (*p* = 0.017).

During zigzag dribbling, the ID and TD groups exhibited statistically significant differences in the total number of right-foot blocks (*p* = 0.031), the total number of left-foot blocks (*p* = 0.001), and the total number of blocks (*p* = 0.003).

### 3.6. Total Number of Steps

The parameters used in the study to measure the total number of steps were the total number of right-foot steps, the total number of left-foot steps, and the total number of steps.

During straight-line dribbling, the ID and TD groups exhibited no significant differences in the total number of right-foot steps (*p* = 0.057), the total number of left-foot steps (*p* = 0.190), and the total number of steps (*p* = 0.075).

During zigzag dribbling, the ID and TD groups exhibited statistically significant differences in the total number of right-foot steps (*p* < 0.001), the total number of left-foot steps (*p* = 0.003), and the total number of steps (*p* < 0.001).

### 3.7. Total Number of Movement

The total number of movements refers to all participants’ movements during the dribbling tests (i.e., kicks, blocks, and steps). The ID and TD groups exhibited statistically significant differences in the total number of movements made during straight-line dribbling (*p* = 0.001) and zigzag dribbling (*p* = 0.002).

### 3.8. Trunk Forward-Backward Angle

**Figure 6:**
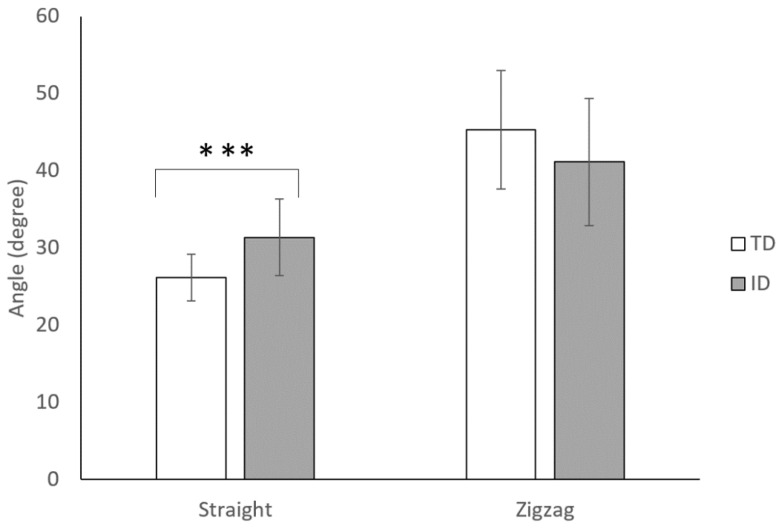
Comparisons of trunk-forward angles of adolescents with TD and ID during the dribbling tests. *** *p*<0.001.

The trunk of all participants mainly tilted forward during soccer dribbling. Figure 6 displays the trunk-forward angles of adolescents with TD and ID during the dribbling tests. In the straight-line test, adolescents with ID exhibited higher trunk forward tilt than those with TD (*p* < 0.001). No significant differences were found in the zigzag test (*p* = 0.059).

### 3.9. Trunk Lateral Sway Angle

**Figure 7:**
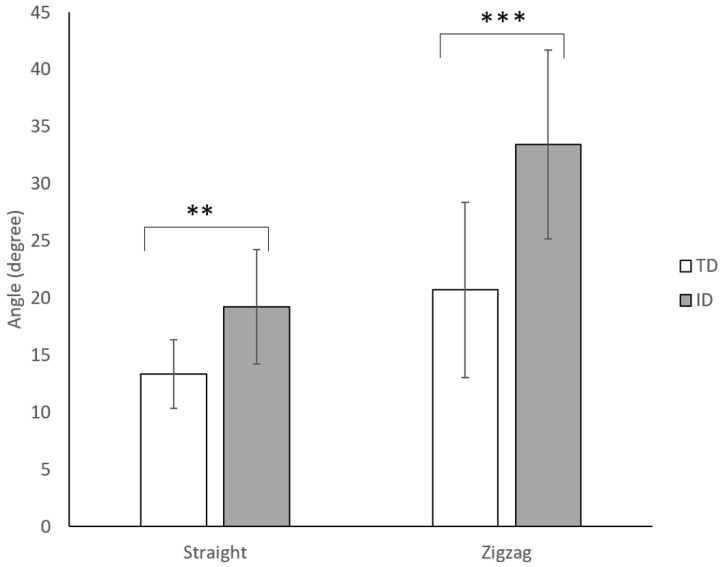
Comparisons of trunk lateral sway of adolescents with TD and ID during the dribbling tests. ** *p*<0.01, *** *p*<0.001.

Figure 7 displays the trunk lateral sway of adolescents with TD and ID during the dribbling tests. Significant differences were found between the two groups in both the straight-line dribbling (*p* < 0.01) and the zigzag dribbling (*p* < 0.001). Adolescents with ID exhibited higher lateral sway in both conditions than those with TD.

## 4. Discussion

By employing customized behavior-coded movement assessment, the lower-limb dribbling parameters of adolescents with ID and their TD peers can be recorded and analyzed quantitatively, accurately, and efficiently. The customized coding parameters were specially chosen for the individuals with ID based on their motor characteristics and level of understanding. The coded features were able to identify and calculate the lower extremity strategic pattern during soccer dribbling. Most of all, adolescents with ID are attracted to soccer, which increases their willingness to perform the requested evaluation or training tasks. The current method is especially suitable for mass screening or follow-up assessment in schools and institutions with individuals with ID. To date, this is the first study that used video-based behavior coding to evaluate and compare the lower extremity performance parameters during dribbling between adolescents with ID and their TD peers.

The results obtained with the flow grid allow us to confirm the hypothesis concerning the differences in dribbling performance between adolescents with ID and TD. The results revealed that a certain degree of movement retardation could be seen in participants with ID. Firstly, in the time domain, significant differences can be found in the total time spent completing the dribbling tasks, both in straight-line and zigzag dribbling. Participants with ID exhibited longer total dribbling time than those with TD. The video recording revealed that the slowness seemed to result from poor balance and coordination. For TD, they dribbled step over step; for ID, they dribbled step by step with less single-leg stance time. Previous studies have indicated that people with ID have relatively poor balance [1,32,33,34] and low physical fitness [35]. Most of them summarized that people with ID had impaired central nervous systems, which leads to weak muscle strength and slower balancing reactions. From our observation, people with ID tended to use strategies that posed fewer fall risks to control the ball. They took smaller steps with fewer arm swings, and their movement seemed stiff and not as synergized as their TD peers. The dribbling movement in TD was quite smooth, rather, the movement in ID was fragmental, and pauses were found occasionally throughout the entire path. The kinematic data from the wearable sensor attached to the back revealed that ID tilted significantly forward compared to their TD peers during straight-line dribbling. As for the more challenging zigzag dribbling, adolescents with ID tended to keep the ball at a low speed. Therefore, less control was needed from their lower limbs. In addition, the trunk was stiff and remained relatively erect in the zigzag dribbling. Data from the lateral sway supported that adolescents with ID swayed significantly and were more variable than their TD peers throughout the exercise. Lateral stability during walking reflects the development of postural stabilization through anticipatory postural actions [36]. Individuals with intellectual disabilities demonstrate a decreased use of feedforward control, which may increase their lateral sway during dribbling.

Secondly, in the frequency domain, participants with ID demonstrated higher numbers of kicks, blocks, steps, and the total number of movements, especially in the challenging zigzag test. Previous studies have shown that people with ID have poorer motor coordination than their TD peers [3,6,18]. As they move, muscle synergy controls the body to remain steady at the proximal ends, which enables the distal ends to achieve various movements, for example, dribbling the ball. However, the different skeletal buildup and the weaker muscle strength limit their control ability. They were less coordinated than their TD peers. Furthermore, participants with ID also demonstrated a higher number of out-of-bounds occurrences, which meant that their ability to control the ball was poorer than TD, thus resulting in more errors. Based on the results, participants with ID did not miss more cones statistically than their TD peers. This can owe to our verbal orders requesting them to go around all cones. As a rule of speed-accuracy tradeoff, individuals with ID needed more time and steps to complete the required task because of their motor control deficiency.

The intention to include both the straight-line and zigzag conditions in the study design was that the difference between TD and ID may not be visible in a straight-line dribble. Nevertheless, the results indicated that the performance differences between individuals with ID and TD were mostly seen in both conditions. The currently available training manuals and research related to soccer dribbling have pointed out that control of the ball during dribbling can be considered in terms of “acceleration”, “increasing speed and distance”, “change of direction”, and “absorption of force” [37,38]. Zigzag dribbling certainly requires more control in the above parameters, but individuals with insufficient motor control (such as those with IDs) pose problems even in straight-line dribbling. Different from a pure kick, straight-line dribbling requires one to have a great deal of control over the speed and acceleration of the ball. In addition, the directional control ability can keep the ball from moving away from their feet and stay along the desired straight line.

The higher numbers of kicks, blocks, steps, and the total number of movements agree with the study findings by Black et al. (2007). They evaluated and compared the treadmill-walking variability of typically developing preadolescents and children with Down syndrome. Their results indicated that the children with Down syndrome partitioned more goal equivalent variance than the typically developing population [39]. Usually, when an individual partitions more goal equivalent variance, it indicates that they have more efficient movement and control. However, compared to TD, the population with ID are not better movers. Black et al. further concluded that children with Down syndrome partitioned more goal equivalent variance because their movement is highly variable. This action allowed children a greater range of solutions to complete the task and therefore allowed them to recover from inefficient movement patterns.

The results further revealed that participants with ID kicked with both feet while dribbling, whereas those with TD mainly used their right foot to kick the ball. Not only in the “kick”. This phenomenon was also found during the “block” event. Since all the participants are right-leg dominant, these indicated that participants with ID did not prefer their dominant leg over the non-dominant one. Kicking with the dominant leg is usually chosen to score the goal [40]. A study by Black et al. (2007) stated that the variability of a movement pattern describes a highly variable system due to a lack of efficient technique. For example, a novice athlete is more varied in the movement to achieve a sport-specific task, while an elite athlete will be more efficient [39]. In this study, the movement pattern of the ID resembled that of a novice athlete who demonstrated immature kicking performance. Because of central nervous system impairments, people with ID often exhibit clumsy and imprecise movements. Studies have shown that because of physical limitations, people with ID are less agile than their typically developing peers [6,41]. They have problems receiving senses, delivering messages, initiating movement output, and an inability to pay attention; consequently, they may pause during movements, thus affecting agility [2,3,8,42]. The findings of this study are consistent with the above-mentioned study results. Still, the kicking and blocking behavior might change if the site is arranged differently, for example wider sidelines or fewer cones. These would result in changes within feet control pattern while dribbling.

This study has several limitations. First, individuals with ID exhibit great individual differences. The current statistical results may represent the motor performance of most IDs, but it cannot be denied that such a number of participants cannot include many variations. Second, these individuals may also demonstrate other clinical symptoms or comorbidities. This study only screened participants based on their motor and cognitive abilities without specifically excluding related comorbidities. Last, many individuals with ID demonstrate impaired cognitive function and sensory integration. The setting of the environment can also be a factor that affects their performance. In this study, the height of the cone, the texture of the floor, and the noise in the field may affect their performance. Since our target participants are more susceptible to related interference than their TD peers, these environmental factors should be controlled rigorously. Future research can address these above-mentioned issues. Since the data collected through the behavior observation software can be stored in the system, appropriate planning can be made for continuously accumulating performance information in this population for big data analysis.

## 5. Conclusions

In contrast to subjective observational recording methods, the customized behavior-coded movement assessment used in this study implemented a scientific approach to define parameters and compare the characteristics of dribbling movements. By effectively recording and calculating the movement parameters of adolescents with ID and their TD peers, the differences between the two groups were clearly identified. The results revealed that adolescents with ID exhibited considerable variances and significantly poorer motor performance than their TD peers in terms of time (longer movement duration) and frequency (increased total number of movements, increased out-of-bounds occurrences). Compared to TD, adolescents with ID also demonstrated immature kicking performance patterns. The customized behavior-coded approach provides an efficient and effective way to gather the movement data and calculate performance parameter statistics in populations with ID. It is especially suitable for mass screening or follow-up assessment in schools and institutions with individuals with ID. Through this approach, lots of manpower can be saved during mass movement screening, making the whole process efficient and effective. Future studies can employ additional testing apparatuses to assess kinetic and kinematic parameters of the limbs, such as joint anthropometric information or muscle strength and endurance.

## Figures and Tables

**Figure 3:**
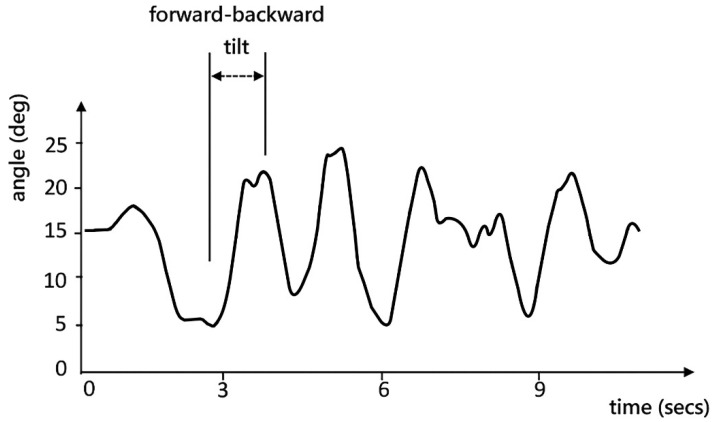
A sample waveform of the trunk forward-backward tilt angle.

**Table 1:** The coded behavior variables.

Source	Parameter	Definition
Video clips	Total Time (s)	The time participants spent dribbling a soccer ball from the start to finish line. The start was defined as the first part of the ball touched the start line. The finish was defined as the last part of the ball crossed over the finish line.
	Number of Out of Bounds	How many times the last part of the ball rolled out the side lines.
	Number of Missed Cones	During zigzag dribbling, the participants must dribble the ball around five cones placed in the mid-line of the site. The number of missed cones refers to how many cones the ball misses as participants dribbled between the whistles signaling start and finish.
	Total Number of Kicks	A kick refers to the action in which a participants’ foot leaves the ground and contacts the ball. The ball and the foot should change in the same direction in at least three consecutive frames. The number of kicks refers to how many times participants kick the ball between the whistles signaling start and finish. The left and right foot was registered separately for further analysis.
	Total Number of Blocks	A block refers to the situation in which the foot contacts the ball as it touches the ground. In at least three consecutive frames the ball changes direction but the foot keeps moving in the same direction. The number of blocks refers to how many times participants block the ball between the whistles signaling start and finish. The left and right foot was registered separately for further analysis.
	Total Number of Steps	A step refers to the situation in which the foot touches the ground but the ball does not change direction and without touching the ball. The number of steps refers to how many times participants step between the whistles signaling start and finish. The left and right foot was registered separately for further analysis.
	Total Number of movements	The total number of movements refers to the sum of all above movements (i.e., kicks, blocks, and steps). The left and right foot was counted separately for further analysis.
Accelerometer	Trunk forward-backward Angle	This angle was defined as the tilt angle of a participant’s trunk on the sagittal plane (forward-backward) during dribbling. A sample waveform of the trunk for-ward-backward tilt angle is depicted in Figure 3. A positive degree indicates forward tilting.
	Trunk lateral sway Angle	This angle was defined as the sway of a participant’s trunk on the frontal plane during dribbling.

**Table 2:** Participants’ Demographics.

	Groups	Mean ± SD	*p*
Physiological age (years)	TD	17.64 ± 0.60	0.561
	ID	16.98 ± 1.05	
Height (cm)	TD	161.75 ± 10.78	0.768
	ID	158.74 ± 11.13	
Gender (M/F)	TD	11/14	0.685
	ID	14/15	

**Table 3:** A coding summary of a participant with ID during zigzag dribbling.

	Time_Absolute_hms	Time_Absolute_ms	Time_Relative_hms	Time_Relative_ms	Time_Relative_s	Duration_s	Obervation	Event_Log	Subject	Behavior	Event_Type
1	14:48:48	109	00:00:04	938	4.938	19.452	E09L03	Event log0001	E01	start	State start
2	14:48:48	610	00:00:05	439	5.439	0.534	E09L03	Event log0001	E01	kickL	State start
3	14:48:49	143	00:00:05	973	5.973	1.535	E09L03	Event log0001	E01	kickR	State start
4	14:48:49	944	00:00:06	773	6.773	7.374	E09L03	Event log0001	E01	blockL	State start
5	14:48:50	678	00:00:07	507	7.507	0.467	E09L03	Event log0001	E01	kickR	State start
6	14:48:51	145	00:00:07	974	7.974	0.634	E09L03	Event log0001	E01	kickL	State start
7	14:48:51	779	00:00:08	608	8.608	1.935	E09L03	Event log0001	E01	kickR	State start
8	14:48:52	814	00:00:09	643	9.643	0.534	E09L03	Event log0001	E01	stepL	State start
9	14:48:53	348	00:00:10	177	10.177	4.771	E09L03	Event log0001	E01	stepR	State start
10	14:48:53	715	00:00:10	544	10.544	0.634	E09L03	Event log0001	E01	kickL	State start
11	14:48:54	348	00:00:11	178	11.178	0.5	E09L03	Event log0001	E01	kickR	State start
12	14:48:54	849	00:00:11	678	11.678	0.667	E09L03	Event log0001	E01	kickL	State start
13	14:48:55	516	00:00:12	345	12.345	0.567	E09L03	Event log0001	E01	kickR	State start
14	14:48:56	84	00:00:12	913	12.913	0.567	E09L03	Event log0001	E01	kickL	State start
15	14:48:56	651	00:00:13	480	13.48	2.302	E09L03	Event log0001	E01	kickR	State start
16	14:48:57	318	00:00:14	147	14.147	2.369	E09L03	Event log0001	E01	blockL	State start
17	14:48:58	119	00:00:14	948	14.948	0.5	E09L03	Event log0001	E01	stepR	State start
18	14:48:58	619	00:00:15	448	15.448	5.439	E09L03	Event log0001	E01	stepL	State start
19	14:48:58	953	00:00:15	782	15.782	1.268	E09L03	Event log0001	E01	kickR	State start
20	14:48:59	687	00:00:16	516	16.516	2.536	E09L03	Event log0001	E01	blockL	State start
21	14:49:00	221	00:00:17	50	17.5	0.534	E09L03	Event log0001	E01	kickR	State start
22	14:49:00	755	00:00:17	584	17.584	0.701	E09L03	Event log0001	E01	kickL	State start
23	14:49:01	455	00:00:18	285	18.285	1.401	E09L03	Event log0001	E01	kickR	State start
24	14:49:02	223	00:00:19	52	19.052	1.201	E09L03	Event log0001	E01	blockL	State start
25	14:49:02	857	00:00:19	686	19.686	2.569	E09L03	Event log0001	E01	kickR	State start
26	14:49:03	424	00:00:20	253	20.253	1.468	E09L03	Event log0001	E01	blockL	State start
27	14:49:04	58	00:00:20	886	20.886	0.567	E09L03	Event log0001	E01	stepR	State start
28	14:49:04	625	00:00:21	454	21.454	8.329	E09L03	Event log0001	E01	stepL	State start
29	14:49:04	892	00:00:21	721	21.721	2.569	E09L03	Event log0001	E01	blockR	State start
30	14:49:05	426	00:00:22	255	22.255	0.634	E09L03	Event log0001	E01	kickL	State start
31	14:49:06	60	00:00:22	889	22.889	0.734	E09L03	Event log0001	E01	kickR	State start
32	14:49:06	794	00:00:23	623	23.623	6.16	E09L03	Event log0001	E01	kickL	State start
33	14:49:07	461	00:00:24	290	24.29	5.493	E09L03	Event log0001	E01	blockR	State start
34	14:49:07	561	00:00:24	391	24.391	5.392	E09L03	Event log0001	E01	stop	State start

**Table 4:** Results for various dribbling parameters.

		Straight-Line		Zigzag	
	Groups	Mean ± SD	t	Mean ± SD	t
Total Time (s)	TD	8.08 ± 2.08	5.269 ***	16.35 ± 4.41	6.153 ***
	ID	12.94 ± 4.77		29.12 ± 10.97	
Number of Out of Bounds	TD	0.11 ± 0.62	4.327 **	0.32 ± 0.46	5.296 ***
	ID	0.19 ± 0.24		1.25 ± 0.95	
Number of Missed Cones	TD	-	-	0.13 ± 0.31	1.903
	ID	-		0.39 ± 0.72	
Total Number of Kicks	TD	4.55 ± 1.33	3.553 **	11.28 ± 2.94	2.092 *
	ID	7.20 ± 3.64		17.68 ± 15.27	
Total Number of Blocks	TD	1.62 ± 1.27	2.279 *	2.55 ± 1.76	2.989 **
	ID	3.40 ± 3.30		6.28 ± 5.53	
Total Number of Steps	TD	13.59 ± 3.18	1.716	20.34 ± 4.83	4.067 ***
	ID	16.72 ± 7.96		34.80 ± 17.02	
Total Number of Movement	TD	19.76 ± 3.51	3.553 **	34.17 ± 5.45	2.320 **
	ID	27.32 ± 9.39		58.76 ± 34.20	
Trunk forward- backward angle	TD	27.64 ± 3.32	−4.723 ***	45.32 ± 7.65	1.929
	ID	33.28 ± 5.577		40.66 ± 6.87	
Trunk lateral sway angle	TD	13.36 ± 4.82	−3.345 **	20.72 ± 6.97	−4.077 ***
	ID	19.24 ± 7.92		33.45 ± 15.04	

***: *p* < 0.001; **: *p* < 0.01; *: *p* < 0.05.

## Data Availability

Not applicable.
